# Salivary Gland Tumors in Pregnancy—Treatment Strategies

**DOI:** 10.3390/jcm14093136

**Published:** 2025-04-30

**Authors:** Małgorzata Wierzbicka, Katarzyna Radomska, Wioletta Pietruszewska, Dominik Stodulski, Bogusław Mikaszewski, Jarosław Markowski, Paweł Burduk, Aldona Woźniak, Jakub Lubiński, Anna Rzepakowska

**Affiliations:** 1 Department of Otolaryngology, Regional Specialist Hospital Wroclaw, Research & Development Centre, 51-124 Wroclaw, Poland; wierzbicka.otolaryngology@gmail.com; 2 Faculty of Medicine, Wroclaw University of Science and Technology, 50-370 Wroclaw, Poland; 3 Institute of Human Genetics, Polish Academy of Sciences, 60-479 Poznan, Poland; 4 Department of Otolaryngology, Pomeranian University of Medicine, 71-252 Szczecin, Poland; 5 Department of Otolaryngology, Head and Neck Oncology, Medical University of Lodz, 90-153 Lodz, Poland; wioletta.pietruszewska@umed.lodz.pl; 6 Department of Otolaryngology, Faculty of Medicine, Medical University of Gdansk, 80-214 Gdansk, Poland; dstodulski@gumed.edu.pl (D.S.); boguslaw.mikaszewski@gumed.edu.pl (B.M.); 7 Department of Laryngology, Faculty of Medical Sciences in Katowice, Medical University of Silesia in Katowice, 40-027 Katowice, Poland; jmarkow1@poczta.onet.pl; 8 Department of Otolaryngology, Phoniatrics and Audiology, Collegium Medicum, Nicolaus Copernicus University, 85-168 Bydgoszcz, Poland; pburduk@cm.umk.pl; 9 Department of Clinical Patomorphology, Poznan University of Medical Sciences, 60-356 Poznan, Poland; 10 Department of Otorhinolaryngology Head and Neck Surgery, Medical University of Warsaw, 02-097 Warsaw, Poland

**Keywords:** salivary gland, tumor, pregnancy, treatment

## Abstract

**Background**: The management of salivary gland tumors (SGTs) during pregnancy is a subject that has received scant attention in the medical literature. While treatment recommendations for cancer therapy in pregnancy have been delineated, those for benign tumors remain unspecified. The present inquiry focuses on the number of women of reproductive age with SGTs and the optimal diagnostic and treatment strategies for tumors occurring during pregnancy. **Materials and Methods**: This was a retrospective multicenter cohort study based on data from the Polish Salivary Network Database, collected between 2018 and 2022. From a total of 2653 patients with salivary gland tumors (SGTs), we identified 1313 women, including 300 of reproductive age (16–42 years). Among them, six cases of SGTs diagnosed during pregnancy were included for detailed analysis. Ethical approval was obtained for this study. **Results**: Among the 300 women of reproductive age, 285 had benign SGTs and 15 had malignant SGTs. Six tumors were diagnosed during pregnancy: four benign (pleomorphic adenomas) and two malignant (salivary duct carcinoma and mucoepidermoid carcinoma). All benign tumors were monitored during pregnancy and surgically treated postpartum. One malignant tumor was resected postpartum, while the second showed a rapid progression in late pregnancy and required early intervention. Individual case details highlighted the diagnostic and therapeutic complexity in this population. **Conclusions**: A standard diagnostic protocol, incorporating ultrasounds and a fine-needle aspiration biopsy, is recommended during pregnancy. For cases in which the clinical and imaging characteristics suggest a benign origin, surveillance is proposed. Conversely, surgical resection is recommended for malignant SGTs, irrespective of the gestational stage. The potential for the malignant transformation of benign tumors during pregnancy in young women underscores the necessity for surgical intervention prior to planned conception.

## 1. Introduction

Cancer complicates approximately 0.1% of all pregnancies, and this incidence continues to increase due to the tendency to postpone childbirth to an older age [1,2]. The most prevalent types of cancer include breast (33%), hematologic (21%), brain (11%), cervical (9%), and ovarian (5%) [3,4]. Melanoma and cancer of an unknown primary origin (CUP) are rare occurrences [5]. The concurrence of malignancy during pregnancy is an infrequent occurrence; however, there is an escalating cognizance that neoplasms can manifest and should be efficaciously managed during this period. The International Network on Cancer, Infertility and Pregnancy (INCIP) has established a registry to document the incidence and maternal, obstetric, oncological, and neonatal outcomes of cancers occurring during pregnancy [6]. The experience in oncological treatment during pregnancy is limited and varies due to the primary location, advancement of the tumor, and stage of pregnancy. Recent studies published in 2023 and 2024 underscore the mounting imperative for clinical guidance on cancer management during pregnancy, particularly in rare localizations such as the salivary glands [7].

The most thoroughly documented treatment outcomes pertain to relatively prevalent neoplasms. Although the diagnosis of breast cancer during pregnancy can be delayed, there is adequate evidence that pregnant women have a 3.1-fold greater chance of being diagnosed with a stage I disease because of frequent doctor examinations [5]. Patients diagnosed with well-differentiated thyroid carcinoma during pregnancy have favorable outcomes for both mothers and children [8]. However, patients diagnosed with cancer of an unknown primary origin (CUP) exhibit a poor response to systemic treatment, with a median maternal survival of eight months [5]. A study of Hodgkin lymphoma patients, both pregnant and non-pregnant, revealed no significant difference in the survival outcomes between these two groups. This finding suggests that antenatal chemotherapy or the deferral of treatment until the postpartum period, with regular obstetric follow-up to ensure fetal growth, may be viable options [9]. The most accurate guidelines for the treatment of cancer during pregnancy were developed for breast cancer [10]. In determining a treatment strategy, it is imperative to consider the patient’s social background, including her family history, pregnancy history, and gestational age. Additionally, the nature of the malignancy and its stage must be taken into account.

The results concerning the fate of newborns from cancer-bearing pregnancies are the subject of separate studies. The available data suggest a correlation between platinum-based chemotherapy and a small size for the gestational age, as well as between taxane chemotherapy and neonatal intensive care unit admission [4]. The management of head and neck cancers during pregnancy is an exceptionally rare occurrence [10,11,12,13,14,15,16,17]. This particular situation necessitates the incorporation of an additional dimension of comprehension—namely, the pregnancy-related aspect—into the existing repertoire of specialized knowledge. This expanded knowledge base should encompass the pathophysiological impact of pregnancy on cancer, the direct and indirect effects of cancer on pregnancy, and the effects of diagnostic and treatment modalities on pregnancy [1,5]. It is imperative to deliberate on the ethical dilemmas associated with decision-making [18]. The concurrence of salivary gland malignancy with pregnancy has been documented in a limited number of cases [1,2,6,12,19,20,21]. The literature on benign salivary gland tumors (SGTs) during pregnancy is particularly limited, with only two cases having been published to date [22,23]. Salivary neoplastic changes are predominantly located in major glands, especially in the parotid gland. A significant proportion of SGTs are of benign origin, with predominant diagnoses of pleomorphic adenoma and adenolymphoma. Malignant SGTs are rare, but the range of histopathological types is broad. The diagnosis of this condition is primarily based on ultrasound imaging and fine-needle aspiration biopsies. Specifically, magnetic resonance imaging is recommended for assessing the involvement of deep neck spaces, and computed tomography is recommended for suspicious bone erosion. The standard therapeutic approach is surgical intervention. The presence of defined pathomorphological features in malignant SGTs, particularly in advanced stages, serves as an indication for the administration of adjuvant radiotherapy.

While recommendations have been established for the timing and methods of treatment in cancers during pregnancy [6], no such recommendations have been established for benign tumors. To address this knowledge gap, an analysis was performed to examine the incidence of SGTs in women of reproductive age on the basis of a large cohort and to reveal all cases of benign and malignant tumors during pregnancy. The exploration of applied treatment strategies was provided with reference to a literature review.

## 2. Materials and Methods

### 2.1. Study Design

The retrospective analysis of patients who developed SGTs during pregnancy has been performed as part of the Polish Salivary Network Database program [24], which comprises 5 years of data (2018–2022) and is an extension of the Polish National Major Salivary Gland Benign Tumors Registry (SGR), which has been in use since 2014 [24]. Six patients whose tumors were diagnosed during pregnancy, out of 2653 SGTs, were identified.

### 2.2. Ethical Approval

The Bioethics Committee at the Karol Marcinkowski Medical University in Poznan, Poland (No. 666/23), approved this study. This project was performed in accordance with the Declaration of Helsinki on biomedical research.

### 2.3. Study Population and Inclusion/Exclusion Criteria

First, the incidence of SGTs among women between 16 and 42 years of reproductive age was analyzed. To further stratify the cohort, women were divided into two subgroups based on age: 16–30 years and 31–42 years. This division reflects demographic patterns in highly developed countries, where women below 30 years are often in the pre-parental planning phase, while those above 30 are more likely to be in the active maternity period. The upper limit of 42 years corresponds to the age of the oldest pregnant patient with a SGT identified in our database. A detailed inspection was subsequently performed in subgroups of young women preparing for parenthood (16–30 years) and women in the maternity period (31–42 years), according to current demographic data from highly developed countries.

### 2.4. Definitions of Variables

Finally, we identified and described 6 cases of SGTs during pregnancy with extremely different clinical courses in the analyzed cohort. Sociodemographic (age and comorbidities) and clinical details (gravidity, diagnosis, tumor detection method, symptoms, tumor histology, treatment features, pregnancy and neonatal outcomes, and the mother’s vital status) were recorded.

Cancers were staged according to the eighth edition of the AJCC/UICC staging system [25]. Histomorphology and immunohistochemistry staging were performed according to the International Agency for Research on Cancer 2017 WHO classification of head and neck tumors, 4th edition [26].

## 3. Results

### 3.1. SGTs in Female Patients

The database included 2653 patients with SGTs, 1313 of whom were women. A total of 300 female patients were identified between 16 and 42 years of age; 285 had benign SGTs, and 15 had malignant tumors. Therefore, the incidence of benign SGTs in women of reproductive age was 10.7%, and the incidence of malignant SGTs was 0.56% in the analyzed cohort. Table 1 presents the distribution of SGTs in different age ranges of the female population. There was an increase in the total number of SGTs with increasing age. There were 3 malignant SGTs and 82 benign SGTs in the 16–30 year age group, whereas in the next age group (31–42 years) there were 12 malignant tumors and 203 tumors.

**Figure 1:**
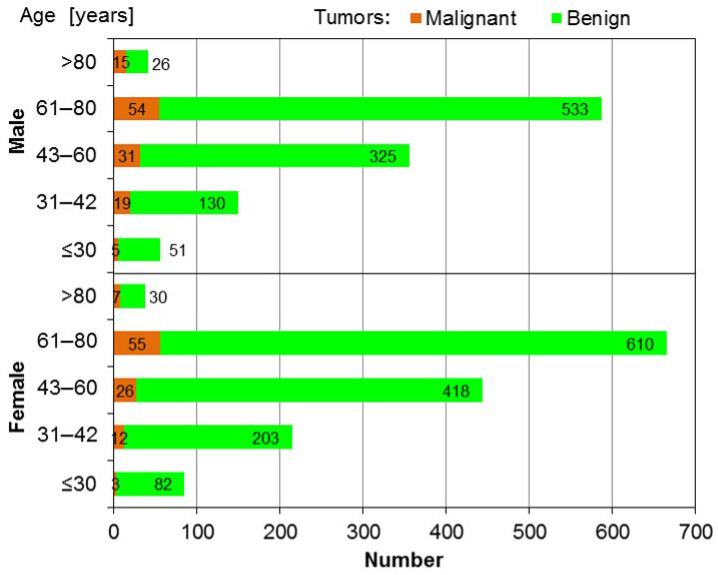
Age and sex distributions of analyzed group of 2653 patients. Disease incidence across age ranges for both sexes.

The age and sex distributions of the 2653 patients included in the analysis are presented in Figure 1.

### 3.2. SGTs in Pregnant Women in the Case Study

A total of six SGTs were diagnosed during pregnancy in the analyzed cohort. Among twelve women aged 31–42 with malignant SGTs, two developed them during pregnancy. Among 285 women with benign SGTs, 4 had a diagnosis during pregnancy and were treated with surgery after delivery.

The following section describes all identified cases of SGTs during pregnancy in detail, with an emphasis on diagnostic difficulties, therapeutic dilemmas, and the legal aspects of medical decisions. A summary is shown in Table 2.

Patient 1 (38 years old)

A 38-year-old woman diagnosed with a parotid tumor showing benign features on imaging planned a parotidectomy, but the procedure was postponed due to the unexpected confirmation of a twin pregnancy. The tumor increased from 3 cm to 4 cm between weeks 12 and 14, then remained stable until delivery. Regular obstetric and parotid ultrasounds were performed. She delivered by cesarean section at 37 weeks without complications. A superficial parotidectomy was performed 6 months postpartum, confirming pleomorphic adenoma. The patient remains disease-free at 5 years.

Patient 2 (28 years old)

A 28-year-old woman, pregnant with her second child, presented at 12 weeks with a 1 cm preauricular mass with features suggestive of pleomorphic adenoma. Despite initial anxiety and pressure for immediate surgery, she agreed to observation with regular ultrasounds. The tumor remained stable throughout pregnancy. She delivered vaginally at 38 weeks. Surgery was performed one month postpartum, and histology confirmed pleomorphic adenoma.

Patient 3 (34 years old)

At 28 weeks of her third pregnancy, a 34-year-old woman was found to have a painless, slightly enlarged parotid mass. An ultrasound showed a well-demarcated lesion with cystic areas. FNAC indicated a benign tumor. She was monitored, and the tumor showed minimal growth. She delivered vaginally at 38 weeks, and a superficial parotidectomy was performed 2 months postpartum. Histopathology confirmed pleomorphic adenoma. She remains disease-free after 2 years.

Patient 4 (34 years old)

A 34-year-old woman noticed a right preauricular lesion at 20 weeks of her first pregnancy. Imaging showed a benign-appearing parotid tumor. The lesion enlarged slightly between weeks 26 and 36. Due to high myopia, a cesarean was performed at 38 weeks. Three months postpartum, a superficial parotidectomy was performed, confirming pleomorphic adenoma. No recurrence has been noted over 3 years.

Patient 5 (33 years old)

A 33-year-old woman presented at 34 weeks of pregnancy with a rapidly growing 6 cm right parotid tumor and facial nerve dysfunction. FNAC revealed malignant cells. Labor was induced at 38 weeks. A total parotidectomy with neck dissection was performed on the fourth postpartum day. Histology confirmed salivary duct carcinoma ex pleomorphic adenoma (T4N3bM0, HER2+, AR–, Ki-67 60–80%). Despite postoperative radiotherapy, brain metastases appeared 3 months later. The patient is alive with disease at the 15-month follow-up.

Patient 6 (32 years old)

A 32-year-old woman presented with a parotid mass at 32 weeks of her second pregnancy. FNAC suggested malignancy. Delivery was scheduled at 38 weeks by cesarean section. Total parotidectomy with selective neck dissection was performed postpartum. Histology confirmed low-grade mucoepidermoid carcinoma (T2N0M0). She remains disease-free at 6 years. Three years later, she was diagnosed with ovarian cancer and was treated successfully.

Treatment strategies for benign and malignant SGTs during pregnancy

The relative indication for surgery is a quickly enlarging tumor of a confirmed benign nature; nevertheless, in this clinical situation, the recommendation should be consistent with any other indication for planned surgery during pregnancy. In addition, the treatment for some very selective malignant cases can potentially be delayed when the disease is well controlled and when the patient wishes to give birth before cancer treatment begins. This applies especially to low-grade malignant SGTs and those in the final trimester of pregnancy when the waiting time for safe delivery is 2–4 weeks.

Patients can refuse surgery even in the optimal trimester of pregnancy if the nodule is benign and undoubtedly stable; however, this is a difficult issue regarding the legal liability of the surgeon.

Relative or absolute indications for surgery during pregnancy include malignant SGTs with a high probability of progression before the planned completion of pregnancy, but of course, this decision ultimately needs to be discussed and accepted by the woman.

**Figure 2:**
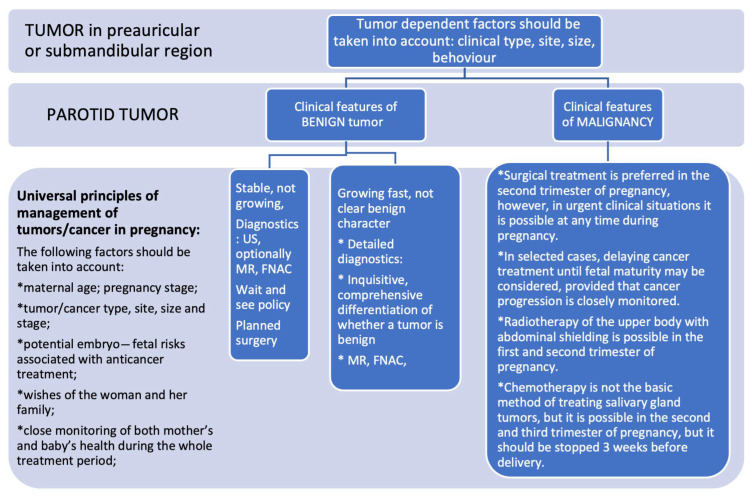
Decision protocol for SGTs in pregnant women.

The suggested decision protocol for SGTs diagnosed during pregnancy is presented in Figure 2.

## 4. Discussion

The present research reveals the issue of SGTs during pregnancy. Among the 2600 SGTs in the database, the number of women of reproductive age was determined, and the calculation indicated a substantial representation of young women among all SGTs. In this cohort, six cases of pregnant women with SGTs with remarkably disparate clinical courses were documented.

These findings raise the question of how to manage a pregnant patient diagnosed with a SGT, taking into account a multitude of factors related to the tumor characteristics, the maternal age, the gestational stage, the patient’s psychosomatic condition, and the patient’s and her family’s preferences. To address this question, we first presented accepted recommendations for cancers during pregnancy; then, we focused on the scarce information for benign tumor management. Finally, on the basis of our own experience and clinical suggestions taken from the literature, the principles for the treatment of SGTs during pregnancy were outlined.

### 4.1. Malignant Tumors

The patient population with malignant SGTs exhibited an absence of a family history of cancer or exposure to risk factors. Risk factors previously identified for developing SDC include prior radiation exposure, occupational exposure to radioactive materials and nickel compounds/alloys, and ambient air pollution from waste gas emissions. A recent study has indicated a potential causal relationship between SDC and Cr (VI) exposure [27] as well as IgG4-related sialadenitis (IgG4-RS) [28]. There is some evidence to suggest that malignant SGTs in women may share risk factors with endometrial, ovarian, and breast cancers, such as early menarche and nulliparity [29]. Additionally, a correlation between the expression of progesterone receptors and the recurrent pleomorphic adenoma of the parotid gland has been observed [30]. However, a subsequent case–control study failed to validate this observation, with the exception of a non-significant correlation between salivary gland cancer and an advanced maternal age at first birth. This association is consistent with the trends observed in endometrial and ovarian cancers [31].

However, the likelihood of identifying a correlation between pregnancy and cancer in a population-based study is also low due to the exceedingly low prevalence of the condition. In the study by Bergaminini et al. [7], which included fifteen pregnant women with head and neck cancers, seven of the patients ultimately succumbed to the disease. In this series, three patients had malignant SGTs (adenocarcinoma, SDC, and myoepithelial cancer). Although two patients received cancer treatment during pregnancy, their survival outcomes were poor. A case of submandibular gland SDC in a young pregnant woman with lung and bone metastases was also examined. Despite multimodal treatment (chemotherapy, radiotherapy, and targeted therapy), the patient died after 36 months due to disease dissemination [21]. Conclusions derived from a limited number of lethal cases are inherently challenging. Despite the fact that the treatment was administered in accordance with contemporary standards and augmented by innovative immunotherapy employing the HER2-targeting monoclonal antibody trastuzumab (Herceptin) [32,33] and a androgen deprivation therapy (ADT) [34], the prognosis for patients with malignant SGTs during pregnancy has proven to be fatal, as demonstrated in studies 34 and 35 [34,35]. A recently published cohort by Bergamini et al. [7] highlighted the poor survival outcomes in pregnant patients with head and neck cancers, including malignant SGTs, despite adherence to multidisciplinary treatment protocols. These findings are consistent with the observations documented in our study, which highlighted the aggressive nature of the tumors and the challenging decision-making scenarios that ensued [7].

As is typical, the onset of symptoms was rapid, and the patient’s tumors were observed to be progressing at an accelerated rate. The decision regarding the continuation or termination of pregnancy in the 35th week was left to the patient and her obstetricians. It is challenging to hypothesize whether these additional four weeks influenced the outcome and brain metastasis development three months after treatment. Our position on this matter is nuanced, aligning with the sentiments expressed by others that the deferral of cancer treatment until fetal maturity is achieved may be contemplated in select cases, contingent upon the meticulous monitoring of the tumor evolution and the maintenance of stability [7,21].

### 4.2. Benign Tumors

A rapid onset and progression during pregnancy is a common feature in the present series and among reported cases [22.23]. In the third trimester of pregnancy, hormonal influences on pleomorphic adenoma growth have been postulated, although this relationship remains unsubstantiated by scientific evidence [35,36]. The prognostic association between the expression of progesterone receptors and the recurrent pleomorphic adenoma of the parotid gland has been previously documented [30], but in our cases, we could not establish such a relationship. The potential for unidentified factors, such as insulin-like growth factor, vascular endothelial growth factor, and human placental lactogen, released by the fetoplacental unit and inducing tumor growth remains a plausible hypothesis [30].

Concomitant with the rapid growth of the tumor, there is a constant concern regarding the potential for malignant transformation and the escalating risk of complications due to the progression of SGTs. In light of the potential for tumor cell dissemination and the limitations of FNAC in discerning the malignancy of SGTs, some authors have opted for surgical resection to mitigate further complications and obtain a definitive diagnosis [23].

### 4.3. Diagnostics and Decision-Making

The extant literature suggests that nonobstetric surgeries can be performed during pregnancy without an elevated risk of adverse outcomes. However, it is imperative to exercise caution when it comes to certain cancer treatments, which should be strategically postponed to the second and third trimesters. This precautionary approach is driven by the heightened risk of fetal harm during the initial three months of pregnancy [37,38]. A systematic literature search of MEDLINE and Scopus was conducted by Haataja et al. The study revealed a decline in the prevalence of nonobstetric surgery over recent decades. However, the rate of scheduled surgery during pregnancy remains at approximately 2 out of 1000 pregnant women, which has been associated with an elevated risk of stillbirth and preterm birth [39].

Benign parotid tumors are not considered emergencies but rather elective surgeries. Consequently, the recommendations for this patient population should align with those for any other indication for planned surgery [40,41]. In the United Kingdom, contemporary guidelines for elective surgery mandate the preoperative establishment of the possibility of pregnancy [42]. It is evident that optimal practices should prioritize patient and fetal safety, necessitating objective documentation prior to any surgical procedure under general anesthetic [43].

Therefore, in pregnant women with SGTs, the following factors should be taken into account when making therapeutic decisions: the clinical characteristics of the tumor and its location, size, and behavior. For clinically benign, stable tumors, a wait-and-see policy based on ultrasound monitoring is recommended, with a subsequent surgical intervention planned postpartum. The presence of ambiguous clinical characteristics necessitates a thorough diagnostic evaluation, particularly in cases of rapid tumor mass growth, as this may indicate malignancy. Tumors of this nature necessitate meticulous diagnostic procedures and the comprehensive differentiation of their characteristics. In the case of pregnant women, staging with imaging tests should be restricted to those associated with the lowest exposure to ionizing radiation. Abdominal plain films, isotope scans, and computerized tomography should be avoided in such cases. Conversely, chest X-rays and ultrasonography are indicated as staging procedures. In select cases of brain tumors or pheochromocytomas, magnetic resonance imaging is recommended, as it has the advantage of avoiding fetal exposure to ionizing radiation [5]. Ultrasonography was the primary imaging modality employed to assess the neck mass in all patients during pregnancy.

This study is not without its limitations. First, the retrospective nature of this study may introduce selection and information biases. Secondly, the relatively small sample size, particularly among the group of pregnant women with SGTs, is attributable to the rarity of the condition, which curtails the statistical power and generalizability of the findings. Thirdly, the diagnostic accuracy of fine-needle aspiration cytology (FNAC) in distinguishing benign from malignant lesions is limited, especially during pregnancy when additional imaging or biopsies may be constrained due to fetal safety concerns. Finally, treatment decisions were often individualized and based on multidisciplinary discussions, which may limit the uniformity of treatment approaches.

In summary, our report emphasized an important feature common to all four cases of benign tumors that revealed a final histological diagnosis of pleomorphic adenoma. Conversely, one of the malignant neoplasms in our cohort originated from a pleomorphic adenoma that underwent malignant transformation. It is plausible that asymptomatic tumors located in the deep part of the parotid gland may have grown slowly for an extended period of time. However, it is important to note that this assertion cannot be unequivocally substantiated, and the impact of pregnancy on the initiation of carcinogenesis remains uncertain. However, the presented case prompted a reflection on the surgical management of symptomatic benign salivary gland tumors in young women prior to planned conception.

This multicenter retrospective study emphasizes the diagnostic and therapeutic complexity of salivary gland tumors during pregnancy. While most benign tumors can be safely monitored until postpartum, malignant lesions may require prompt intervention. The observed risk of malignant transformation, even in tumors previously considered benign, supports the recommendation for surgical resection before planned conception in selected cases.

Given the rarity of SGTs during pregnancy, there is a pressing need for the creation of prospective, multi-institutional and international registries. Such collaborative efforts would allow for the accumulation of standardized data, enable comparative studies, and support the development of evidence-based clinical guidelines for both benign and malignant salivary gland tumors diagnosed during pregnancy.

## Figures and Tables

**Table 1:** The percentages of women in the selected age cohorts in relation to the entire group of 2653 patients were divided into benign and malignant tumors.

	Malignant Tumors		Benign Tumors	
Age	n	%	n	%
16–30	3	0.113%	82	3.091%
31–42	12	0.452%	203	7.652%
16–42	15	0.565%	285	10.743%

**Table 2:** Characteristics of women with salivary gland tumors during pregnancy.

No	Age	Gravida	Familyl History of Cancer	Gestational Age at Diagnosis	Tumor Growth During Pregnancy	Delivery	Histology (HP)	Tumor Size (cm)	Timing of Surgery	Final Diagnosis	DFS/Outcome	Special Notes
1	38	1	No	Pre-pregnancy (surgery delayed)	Stable	C-section (twains)	FNAC Milano III	4.0	6 months postpartum	PA	5 years/Vital	Infertility treatment
2	28	2	Yes	12 weeks	Enlarged (12–16 w), then stable	Natural at 38 wks	FNAC Milano III	3.0	1 months postpartum	PA	Not-reported/Vital	Civil lawsuit withdraw
3.	34	3	No	28 weeks;	Slightly enlarged	Natural at 38 wks	FNAC Milano III	3.0	2 months postpartum	PA	2 years/Vital	Factor VII mutation
4.	34	1	No	20 weeks	Gradual growth	C-section at 38 wks	FNAC Benign cells	2.0	3 months postpartum	PA	3 years/Vital	myopia
5.	33	1	Mother breast cancer	34 weeks	Rapid growth (doubling)	Induced at 38 wks	FNAC Cancer cells	6.0	4 months postpartum	SDC ex PA (T4N3bM0)	15 months, Alive with metastases	HER2+, AR-, Ki67 60–80%
6	32	2	Ovarian cancer (pt)+leukemia (child)	32 weeks	Not specified	C-section at 38 wks	FNAC–malignant cells	3.0	postpartum	Mucoepidermoid carcinoma (low grade)	6 years/Vital	Later ovarian cancer

HP—histopathology results; PA—pleomorphic adenoma; FNAC—fine-needle aspiration cytopathology; US—ultrasound; SDC—salivary duct cancer; and DSF—disease-free survival.

## Data Availability

Data, not contained in the manuscript, are available on special request from first author.
